# Effect of position and exercise on measurement of muscle quantity and quality: towards a standardised pragmatic protocol for clinical practice

**DOI:** 10.1186/s13102-020-00227-3

**Published:** 2021-01-07

**Authors:** Carly Welch, Zeinab Majid, Isabelle Andrews, Zaki Hassan-Smith, Vicky Kamwa, Hannah Picton, Daisy Wilson, Thomas A. Jackson

**Affiliations:** 1 MRC-Versus Arthritis Centre for Musculoskeletal Ageing Research, University of Birmingham and University of Nottingham, Birmingham, UK; 2 Institute of Inflammation and Ageing, University of Birmingham, Birmingham, B15 2TT UK; 3 University Hospitals Birmingham NHS Foundation Trust, Birmingham, B15 2GW UK; 4 Musculoskeletal Endocrinology Research Group, Centre for Endocrinology, Diabetes and Metabolism, Birmingham Health Partners, Birmingham, UK

**Keywords:** Sarcopenia, Diagnostic imaging, Electric impedance, Muscles

## Abstract

**Background:**

Ultrasonography is an emerging non-invasive bedside tool for muscle quantity/quality assessment; Bioelectrical Impedance Analysis (BIA) is an alternative non-invasive bedside measure of body composition, recommended for evaluation of sarcopenia in clinical practice. We set out to assess impact of position and exercise upon measures towards protocol standardisation.

**Methods:**

Healthy volunteers aged 18–35 were recruited. Bilateral Anterior Thigh Thickness (BATT; rectus femoris and vastus intermedius), BATT: Subcutaneous Ratio (BATT:SCR), and rectus femoris echogenicity were measured using ultrasound and BIA was performed; 1) lying with upper body at 45° (Reclined), 2) lying fully supine at 180^o^ (Supine), 3) sat in a chair with upper body at 90^o^ (Sitting), and 4) after exercise Reclined. Variability of Skeletal Muscle Mass (SMM) by two different equations from BIA (SMM-Janssen, SMM-Sergi), phase angle, fat percentage, and total body (TBW), extracellular (ECW), and intracellular water (ICW) were assessed.

**Results:**

Forty-four participants (52% female; mean 25.7 years-old (SD 5.0)) were recruited. BATT increased from Reclined to Sitting (+ 1.45 cm, 1.27–1.63), and after exercise (+ 0.51, 0.29–0.73). Echogenicity reduced from Reclined to Sitting (− 2.1, − 3.9 – -0.26). SMM-Sergi declined from Reclined to Supine (− 0.65 kg, − 1.08 – − 0.23) and after exercise (− 0.70 kg, − 1.27 – -0.14). ECW increased from Reclined to Sitting (+ 1.19 L, 0.04–2.35). There were no other statistically significant changes.

**Conclusion:**

Standardisation of protocols is especially important for assessment of muscle quantity by ultrasonography; BIA measurements may also vary dependent on the equations used. Where possible, participants should be rested prior to muscle ultrasonography and BIA, and flexion of the knees should be avoided.

## Background

Sarcopenia is a condition of increasingly recognised significance in research and clinical practice. It is defined as reduced muscle strength with reduced muscle quality and/or quantity, and associated with significant detriments in quality of life and adverse health outcomes [1]. Dual-energy X-ray Absorptiometry (DXA), Computed Tomography (CT), and Magnetic Resonance Imaging (MRI) are recommended as Gold Standard for muscle quantity measurement [1], but these are time-consuming, cannot be performed at the bedside, and are rarely performed serially. Ultrasonography is an emerging tool for assessment of muscle quantity and quality as part of evaluation for sarcopenia [2–4]. It has evident benefits in that it is non-invasive, without exposure to ionising radiation, and provides point of care measurement in a number of settings. However, there is a lack of agreement on how muscle ultrasonography protocols for sarcopenia assessment should be standardised across clinical settings, including participant position and rest requirements pre-procedure [5, 6]. Bioelectrical impedance analysis (BIA) is an alternative safe technique for assessment of muscle quantity; phase angle, a direct measure of the angle between resistive current and total current, has been proposed as a measure of muscle quality by BIA [7]. Higher values suggest greater cellularity and cell membrane integrity. The use of BIA has been criticised in research settings, due to reduced accuracy compared to DXA, CT, and MRI [8]. However, it may be a pragmatic tool in clinical practice for body composition estimation [1]; it can be performed within minutes at the bedside, with minimal training. This study set out to evaluate the effect of changes in position and exercise upon muscle quality and quantity measured using ultrasound and BIA, in order to demonstrate the validity and recommendations of either or both techniques for use in clinical practice.

## Methods

### Participants

Healthy young adults aged 18 to 35 were recruited to this study at the University of Birmingham Research Laboratories, Queen Elizabeth Hospital Birmingham, in February 2020. Ethical approval was obtained from the University of Birmingham Science, Technology, Engineering and Mathematics Ethical Review Committee (ERN_19–1173). All study participants provided written informed consent to participate in this study. Exclusion criteria were: acute or chronic infectious or inflammatory conditions, inability to mobilise independently without walking aids, and the use of immunosuppressive agents or systemic steroids. Data were collected on age, sex, ethnicity, and physical activity via the Global Physical Activity Questionnaire [9]. Handgrip strength, gait speed over four metre course, height, and weight were measured for all participants.

### Ultrasonography

Bilateral Anterior Thigh Thickness (BATT) was measured as described previously [3] with B-mode ultrasonography using a linear probe (Venue 50, GE Healthcare). A mark was made on the skin at the midpoint between the greater trochanter and the lateral joint line of the knee on both sides and all measurements were taken at this mark. Participants were advised to relax their muscles. Contact gel was applied to the skin. The rectus femoris (RF) was identified by locating its border, and the probe was positioned in the transverse plane so that the RF was central over the femur. The thickness of subcutaneous tissue (SC), RF, and vastus intermedius (VI) were measured in real time at central point of greatest thickness, with the probe held in maximal relaxation, to a depth of 7 cm. If it was not possible to view the entire VI, the minimum visible thickness was used in analysis. A minimum of three measurements were taken on each side; a fourth was taken if measurements differed by more than 10%. The mean of all measurements on each side was calculated and used in analysis. Bilateral Anterior Thigh Thickness was calculated as the total of right (RF + VI) + left (RF + VI). BATT:SC ratio (BATT:SCR) was calculated by dividing BATT by total bilateral SC. This method has been shown to have excellent intra-rater and inter-rater variability when using the same protocol [3]. All images for individual participants were taken by the same sonographer. All images were saved and remotely checked by a second experienced sonographer to ensure satisfactory views and measurements had been obtained. A further image was taken in the longitudinal plane and RF grey scale analysis was performed using Image J software [3]. Grey scale analysis was calculated by drawing a square within the RF and analysing within this section. A measure of between 0 (black) and 255 (white) was returned. Echogenicity was calculated as the mean RF grey scale from both sides. This provides a measure of muscle quality and is considered to correlate with intramuscular fat infiltration [3].

### Bioelectrical impedance analysis

Impedance was measured using a Bodystat Quadscan 4000. Electrodes were placed on the right hand and foot as per the manufacturer’s instructions and connected to the device. Height, weight, and age were inputted into the device and readings were then generated. All readings were recorded in real time including impedance, resistance, reactance, phase angle, fat percentage, total body water (TBW), extracellular water (ECW), and intracellular water (ICW). These are readings that are provided directly from the device using internal calculations. The phase angle equation is shown in Table 1.Skeletal muscle mass (SMM) was calculated using two widely accepted calculations – SMM-Janssen [10] and SMM-Sergi [11], as shown in Table 1.

**Table 1:** Equations used in calculation of Skeletal Muscle Mass (SMM) using bioelectrical impedance analysis. *In both equations: Height in cm; Sex 1 = male, 0 = female; Weight in kg; Resistance in Ω; Reactance in Ω*

Skeletal Muscle Parameter	Equation
SMM-Sergi [11]	= −3.964 + [0.227 × (height^2^/resistance)] + (0.095 × weight) + (1.384 × Sex) + (0.064 × reactance)
SMM-Janssen [10]	= [(height^2^/resistance) × 0.401] + (Sex × 0.3825) + (Age × − 0.071) + 5.102
Phase angle	= arctan (reactance/resistance) *“arctan” is the inverse trigonomic function (arc tangent) of the tangent function*

### Positions and exercise protocol

**Fig. 1:**
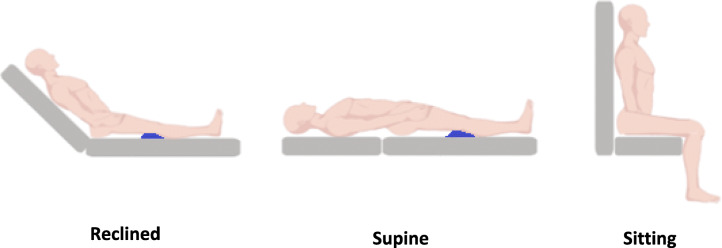
Positions utilised during study. In the Reclined position participants were positioned at 45^o^ with a wedge below their knees, in the Supine position participants were positioned supine with a wedge below their knees. In the Sitting position participants were sat upright in a chair

Initial BIA and ultrasound measurements were taken following a period of rest with the participant positioned lying on a couch, with their upper body at 45^o^ and a firm wedge placed below their knees (Reclined). Measurements were then repeated with the participant lying flat at 180^o^ with the same wedge (Supine), and sat in a chair at 90^o^ (Sitting). The chair and couch used were of a similar firmness. Participants were advised to complete 20 star-jumps, 20 squats, and 20 burpees, or until they tired (Additional file 1). Measurements were then repeated immediately in the Reclined position. Figure 1 shows the different positions that were utilised. The same order of measurements was used for all participants.

### Statistical analysis

Data were imported into IBM SPSS Statistics 26 for analysis. Descriptive statistics are represented in text and tables. Normalities of outcomes were assessed visually using Q-Q plots and histograms, and statistically by Shapiro-Wilk tests. Where outcomes were normally distributed, differences across positions and after exercise were assessed using linear mixed models to account for missing data. If not normally distributed, differences were assessed using generalised linear mixed models.

## Results

### Participants

Forty-four participants were recruited; mean age 25.7 (SD 5.0), 52% female. Full participant characteristics are shown in Table 2. Missing data and participant inclusion for each position, including data excluded on second review, are shown in the online supplement (Additional file 1).

**Table 2:** Participant characteristics

		Study population (*N* = 45)
Age (years) – mean (SD)		25.7 (5.0)
Gender – Female % (N)		52% (23)
Ethnicity	Black or Black Mixed % (N)	34% (15)
	East Asian or Mixed East Asian % (N)	14% (6)
	South Asian or Mixed South Asian % (N)	52% (23)
METminutes/week – mean (SD)		3436 (2790)
Sedentary minutes/week – mean (SD)		2876 (1308)
Meeting recommended activity – % (N)		98 (43)
Body Mass Index (kg/m^2^) – mean (SD)		23.4 (4.0)
Handgrip strength (kg) – mean (SD)	Males	56.8 (12.5)
	Females	32.3 (4.6)
Gait speed (m/s) – mean (SD)		1.38 (0.26)

METminute = Metabolic Equivalent minutes METminutes were calculated from the Global Physical Activity Questionnaire as the sum of weekly vigorous (minutes × 8) and moderate (minutes × 4) activities performed as part of work, commuting, and leisure. Physical activity cut-off of < 600 METminutes/week was considered as not meeting recommendations [9]

### Ultrasonography

#### Bilateral anterior thigh thickness (BATT)

**Fig. 2:**
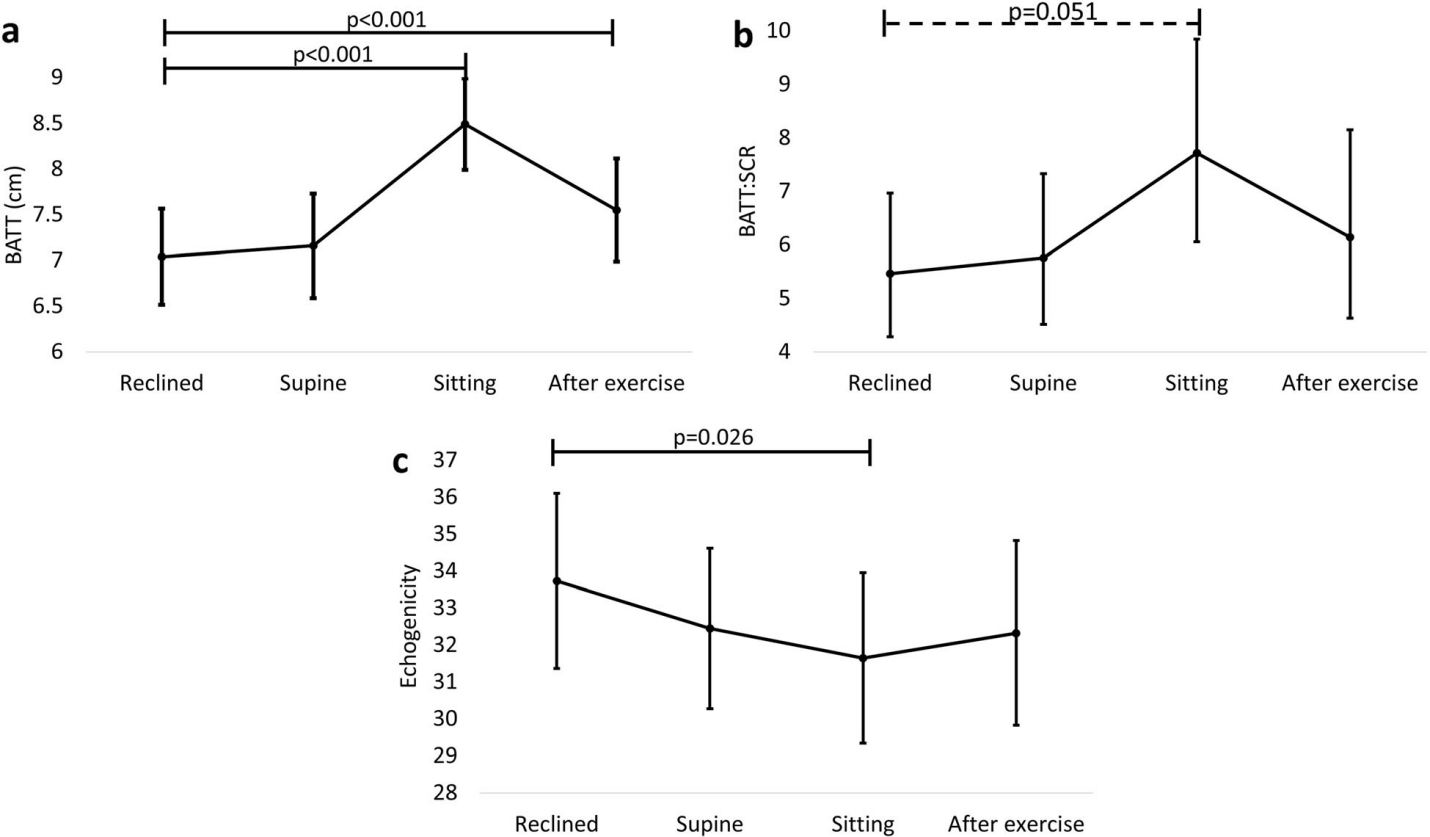
Differences in ultrasonography measures between positions and after exercise. Markers correspond to estimated means calculated from linear mixed models/ generalised linear models. Error bars correspond to 95% confidence intervals. BATT = Bilateral Anterior Thigh Thickness; BATT:SCR = Bilateral Anterior Thigh Thickness: Subcutaneous tissue Ratio

The RF and VI were measured in all patients in Reclined and Supine positions. However, the VI could not be fully visualised Sitting in 9.8% (4/41) of participants; in these cases, BATT was calculated from the visible VI thickness. Bilateral Anterior Thigh Thickness increased from Reclined to Sitting (+ 1.44 cm, 1.27–1.63; *p* < 0.001), and after exercise (+ 0.51, 0.29–0.73; *p <* 0.001) (Fig. 2a). There was no statistically significant change from Reclined to Supine. Variations in individual participant data are shown in the online supplement (Additional file 1).

#### Bilateral anterior thigh thickness: subcutaneous ratio (BATT:SCR)

Bilateral Anterior Thigh Thickness: Subcutaneous tissue Ratio did not significantly differ between positions but there was a trend towards decline from Reclined to Sitting (− 2.26, − 4.53 – + 0.01; *p* = 0.051) (Fig. 2b).

#### Echogenicity

Echogenicity reduced from Reclined to Sitting (− 2.1, CI -3.9 – − 0.3; *p* = 0.026), but other changes were not statistically significant (Fig. 2c).

### Bioelectrical impedance analysis

#### Skeletal muscle mass

**Fig. 3:**
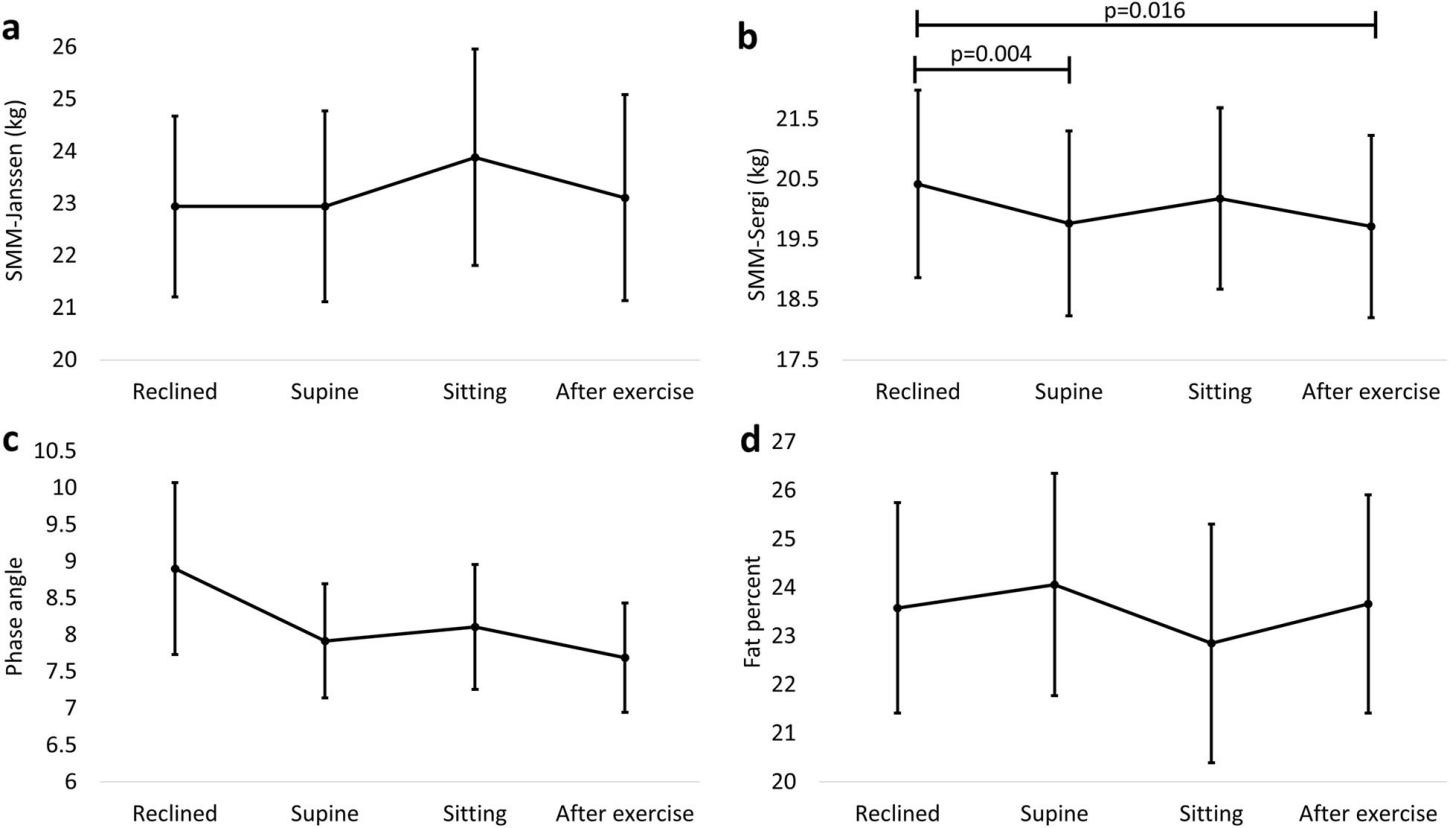
Differences in muscle and fat measures by bioelectrical impedance analysis before and after exercise. Markers correspond to estimated means calculated from linear mixed models/ generalised linear models. Error bars correspond to 95% confidence intervals. SMM-Janssen = Skeletal Muscle Mass by Janssen equation; SMM-Sergi = Skeletal Muscle Mass by Sergi equation

SMM-Janssen did not differ significantly between positions or after exercise (Fig. 3a). SMM-Sergi reduced from Reclined to Supine (− 0.65, CI -1.08 – − 0.23; *p* = 0.004) and after exercise (− 0.70, CI -1.27 – − 0.14; *p* = 0.016) (Fig. 3b).

#### Phase angle

Phase angle did not statistically significantly differ between positions or after exercise (Fig. 3c).

#### Fat percentage

Fat percentage did not differ significantly between positions or after exercise (Fig. 3d).

#### Water distribution

**Fig. 4:**
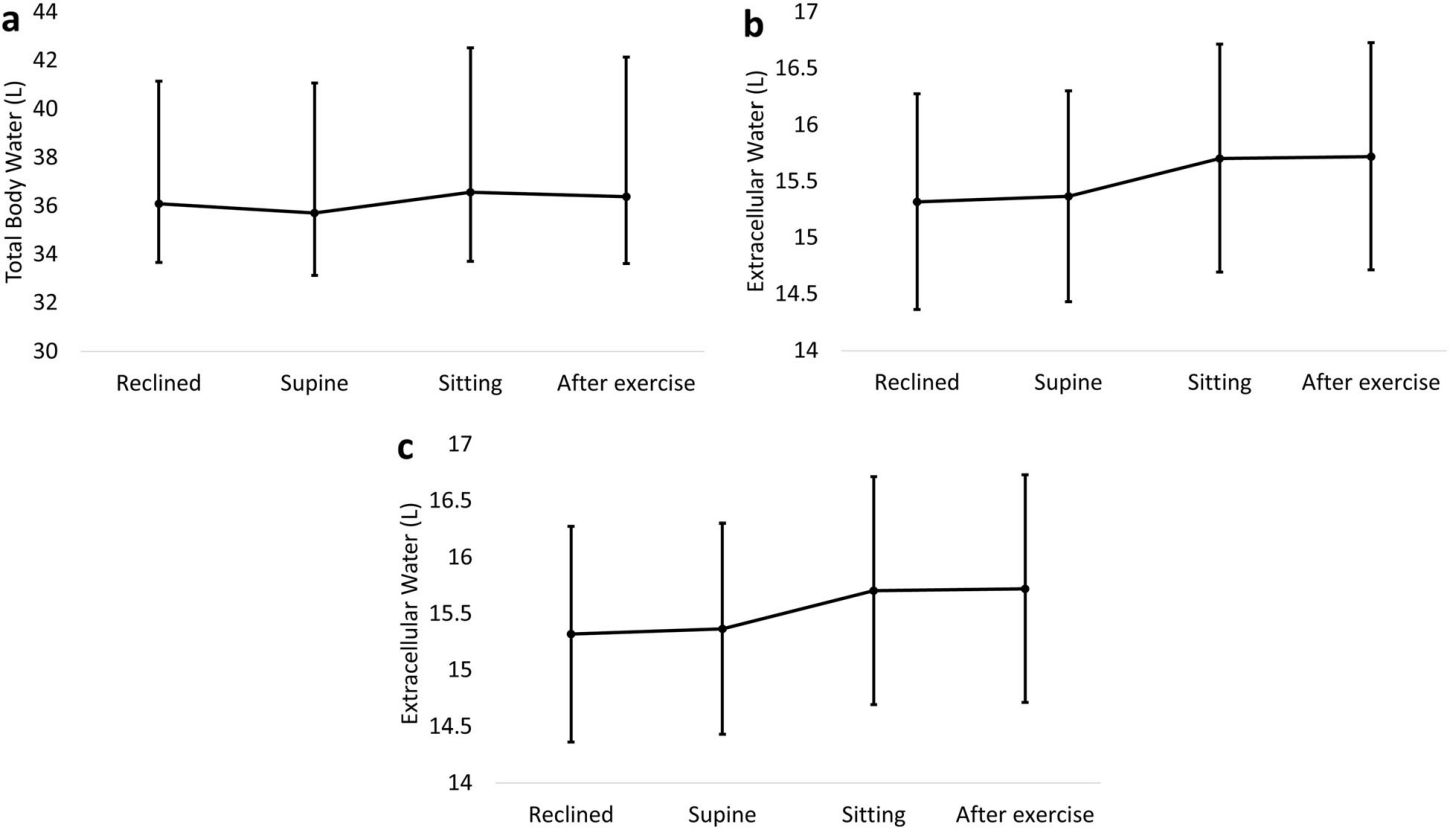
Differences in water distribution measurements by bioelectrical impedance analysis. Markers correspond to estimated means calculated from linear mixed models/ generalised linear models. Error bars correspond to 95% confidence intervals

TBW, ECW, and ICW did not significantly differ between positions or after exercise (Fig. 4a-c).

## Discussion

### Interpretation and implications for future research and clinical practice

Bilateral Anterior Thigh Thickness exhibited the greatest variance in relation to both position and exercise, the greatest of which was the effect of sitting in a chair. Increases in BATT were exhibited when participants were sat in a chair as compared to measurements performed on the examination couch. This is consistent with previous studies, which have shown increased RF cross-sectional area in the seated position compared to supine [12]. As it was not possible to view the entire VI in all participants in this position, the demonstrated effect is likely to be an underestimate and the true difference may be even greater. To a lesser degree, BATT also increased after exercise. The exercise protocol used in this study was more intensive than a typical exercise protocol that might be used in a frail or hospitalised population. However, even small increments in physical activity could be equivalently demanding in people with frailty or acute illness; for an older frail person this could be simply the demand of walking across a room and getting onto an examination couch. Bilateral Anterior Thigh Thickness has been shown to have excellent intra-rater, inter-rater variability when using the same protocol (i.e. repeated measures in the same position); we are confident that these changes relate to the effect of position and exercise. Additionally, validity of measurements was ensured by review of all by a second experienced sonographer, including correct orientation of the RF over the femur.

Importantly, the difference in BATT between the recumbent and sitting positions (+ 1.44 cm) was greater than differences that have been observed in clinical studies measuring changes in muscle quantity in hospitalised populations [13] i.e. highly clinically significant. This difference is also greater than the 95% confidence intervals for estimated mean BATT in all positions. It is important to consider that the differences in BATT do not relate to true differences in muscle quantity within these short time frames. Increased BATT in the seated position likely relates to contraction and shortening of the RF with combined knee and hip flexion, leading to a greater cross-sectional area; the RF inserts at both the hip and knee joint [12]. As it is not possible to measure muscle volume with ultrasonography, this emphasises why standardisation of protocols is vitally important. Similarly, BATT increased after exercise, likely related to persistent contraction of the quadriceps muscles. During exercise, metabolic requirements of skeletal muscles are increased and blood flow increases [14]. This in turn increases the temperature of muscles and reduces stiffness, promoting increased muscle activity i.e. muscle contraction in the neutral position.

Echogenicity declined in the seated position, but there were no significant changes after exercise. Additionally, the change in the seated position was smaller and potentially of less clinical significance. Echogenicity provides a numerical measure of muscle quality, which has been shown to correlate with muscle function [3]. Therefore, echogenicity may provide a more readily standardisable measure across settings, where standardisation of exercise protocols is challenging. However, echogenicity has been shown to exhibit greater inter-user variability compared to BATT [3]. As all images for individual participants were obtained by the same sonographer, this should not have affected changes demonstrated across repeated measures for individual participants.

As much as possible, position should be standardised when performing quadriceps muscle ultrasonography; where there are any deviations in position, these should be noted. The seated position may represent an option as a pragmatic, easily standardised position. However, as we were unable to obtain thickness measurements in all patients in this position, this may be less feasible without readily available machines/probes that measure to greater depth. This is important when measuring healthy young adults as part of a reference standard, but may also be particularly relevant in individuals with increased subcutaneous tissue e.g. sarcopenic obesity. We recommend that ultrasonography measures should be taken with the knee in natural relaxation. As we did not find any clinically or statistically significant difference between the supine and 45^o^ positions, small variations in the tilt of the head of the bed can be tolerated, provided significant flexion of the knee is avoided.

Less variance was exhibited with BIA. Phase angle, SMM-Janssen, fat percentage, TBW, ECW, and ICW did not vary across any repeated measures statistically significantly. There were reductions in SMM-Sergi from the 45^o^ position to fully supine and after exercise. Pragmatically, this means that BIA can be performed in a variety of clinical settings, including where it is not practical to perform supine e.g. in a frail older person attending a clinic appointment in a wheelchair. A more reliable formula where the position of the upper body cannot be standardised but the patient/participant is able to lie on a couch or a period of rest prior to assessment is not feasible may be SMM-Janssen. Historically, BIA has been extensively criticised previously compared to DXA, CT or MRI in research settings, due to reduced precision [8]. However, it is also important to consider the purpose of measuring muscle quantity and the degree of certainty that is necessary in clinical practice. BIA may be a pragmatic tool for screening and as an adjunct as part of a Comprehensive Geriatric Assessment. As well as less variability demonstrated in this study with positions and exercise, BIA is also much quicker to perform than ultrasound and requires minimal training. The phase angle has been proposed as a measure of muscle quality, as a measure of cell membrane function [7]. However, BIA is known to be affected by fluid balance [4], although as technology and datasets develop it may be possible to perform correction calculations for this. BIA is also currently contraindicated in people with implanted cardiac devices; there is increasing evidence that it is likely to be safe [15], but it is unknown if results can be reliably interpreted.

### What are the limitations of this research?

Importantly, this research was performed in healthy young volunteers. Whilst our results provide preliminary results towards standardisation of a protocol for muscle quantity assessment, we recognise that results may be different in an older and/or hospitalised population. In older adults with sarcopenia, less variability in measures may be seen if muscles are already very small and insufficient. Indeed, a pragmatic interpretation may be that if muscle quantity is demonstrated to be reduced in the seated position, then it is very likely to be reduced in any other position. However, if muscle quantity appears normal it may still be reduced if measured without the hip and knee in combined flexion.

Conversely, in hospitalised populations it is plausible that greater variability in measures may be exhibited due to greater fluid shifts. This may affect measurements taken using ultrasonography as well as BIA. In our study, all participants were young, healthy, and clinically euvolaemic. There was no clinical evidence of change in hydration status between repeated measures, and hydration status measured by BIA itself also did not change with position. Additionally, nearly all participants were sufficiently physically active to meet the minimum World Health Organization (WHO) guidelines, which may have affected the responsiveness of skeletal muscles to the effects of position and exercise. Our study was not powered to examine differences of position and exercise effect between groups (e.g. gender, ethnicity, activity levels). However, since participant characteristics did not change between repeated assessments, this will not have affected our overall results.

Whilst we consider the changes in BATT and BATT:SCR not to be related to true changes in muscle quantity, we recognise that we did not measure muscle quantity using any gold standard techniques. Due to the nature of the study, it was also not possible to blind assessors to position. Additionally, considering the effects of exercise, this study only evaluated the effects of very short high intensity exercise; the effects of longer periods of exercise, or less intensive physical activity are unknown. We also acknowledge that we cannot rule out effects of moving between positions in the order used, as we did not use a counterbalance design.

## Conclusion

Measured muscle quantity, but not quality, varied by ultrasonography with changes in position and after exercise in this study involving healthy young volunteers. Muscle quantity measurements using BIA were not affected by position or exercise. Further research evaluating these changes in older adults will be valuable. However, as cut-off values for the diagnosis of sarcopenia are developed from young healthy reference populations (9), we consider it important to standardise technique in these populations to ensure measures taken in clinical populations are comparable.

We recommend that ultrasonography measures should be taken with patients/participants resting on a bed/couch with knees in natural extension. Whilst prolonged periods of rest may not be practical, patients/participants should avoid exertion immediately before muscle ultrasonography; we recommend measuring physical performance afterwards. When deciding on appropriate tools for assessment, it is important to consider the purpose for muscle quantity/quality measurements. For screening purposes, then BIA may be suitable. If the purpose is for more comprehensive evaluation, then ultrasonography and BIA can be performed together as part of a comprehensive assessment e.g. to test responsiveness to interventions.

## Supplementary Information

**Additional file 1.**

## Data Availability

The datasets used and analysed during the current study available from the corresponding author on reasonable request.

## Abbreviations

BATT: Bilateral Anterior Thigh Thickness
BATT:SCR: Bilateral Anterior Thigh Thickness: Subcutaneous tissue Ratio
BIA: Bioelectrical Impedance Analysis
CT: Computed Tomography
DXA: Dual-energy X-ray Absorptiometry
ECW: Extracellular Water
ICW: Intracellular Water
MRI: Magnetic Resonance Imaging
RF: Rectus femoris
SC: Subcutaneous
SD: Standard Deviation
SMM: Skeletal Muscle Mass
SMM-Janssen: Skeletal Muscle Mass (Janssen equation)
SMM-Sergi: Skeletal Muscle Mass (Sergi equation)
TBW: Total Body Water
VI: Vastus intermedius
