# Supplementary material for: Effect of position and exercise on measurement of muscle quantity and quality: towards a standardised pragmatic protocol for clinical practice
Source: BMC Sports Sci Med Rehabil. 2021 Jan 7;13:3. doi: 10.1186/s13102-020-00227-3 (PMC7792326; doi:10.1186/s13102-020-00227-3)
Supplement: Supplementary file 1 — Additional file 1. [file 13102_2020_227_MOESM1_ESM.docx]

# Supplementary data

## Appendix 1 – Exercise protocol

Participants were advised to complete the following exercises:

- 20 Star-jumps: Participants were advised to jump from a standing position with their arms by their side, to a wide legged stance with their legs separated and their arms in a “V” position above their heads. This was achieved through shoulder abduction and moving their arms sideways into the posture. Participants then jumped back into their original resting posture with their arms by their sides and their feet together in parallel. They were asked to repeat this 20 times.
- 20 Squats: Participants were advised to stand with their feet shoulder width apart and squat down by bending their knees while moving their hips back, until their hips were parallel to or just below their knees. They were then advised the push themselves back to the standing position, keeping their knees and chest out, whilst pulling their hips up. They were asked to repeat this 20 times.
- 20 Burpees: Participants were advised to start in neutral standing position with their hands by their sides. They were then advised to squat down with their knees fully bent and their hands touching the floor, and from this position immediately push themselves forwards into a “plank” or “push-up” position. In this position, the toes remained touching the floor, and their body was flat but not touching the floor, with their hands on the floor underneath their shoulders, and their arms bent at the elbows. They were then advised to move back into the full squat position in a single movement, and from this position to jump up back into the standing position with their hands above their head. There asked to repeat this 20 times, or until they were unable to complete further burpees due to muscle soreness or fatigue.

## Appendix 2 – Missing data for each position/ repeated measure


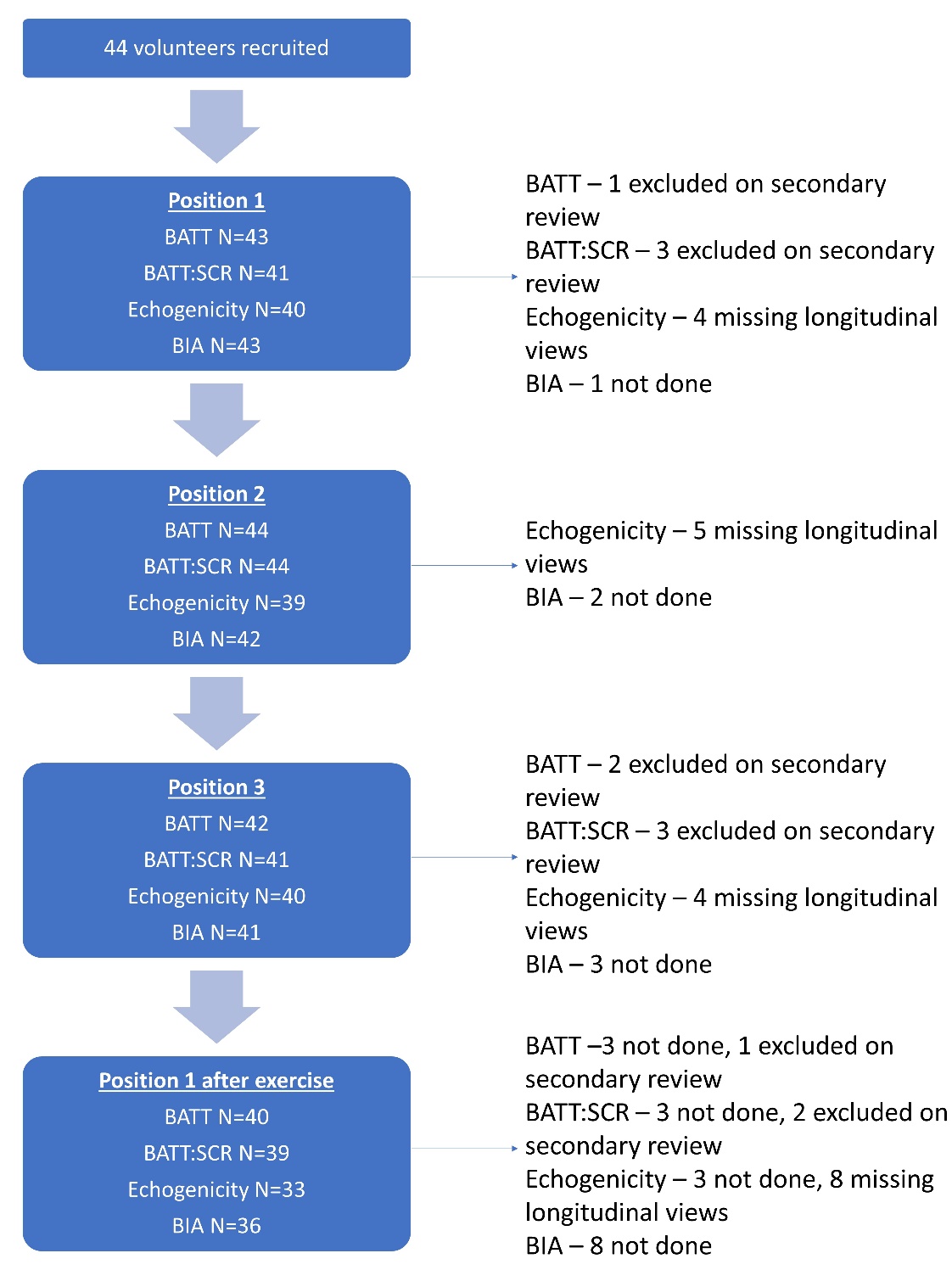


## Appendix 3 – Individual participant changes with position and after exercise
